# Ganciclovir penetrates into the cerebrospinal fluid of an infant with congenital cytomegalovirus infection

**DOI:** 10.1186/s13052-015-0132-8

**Published:** 2015-03-31

**Authors:** Fabio Natale, Bianca Bizzarri, Veronica Cardi, Aurelia Gaeta, Paola Villani, Giuseppina Liuzzi, Mario De Curtis

**Affiliations:** Department of Pediatrics and Child Neuropsychiatry, “La Sapienza” University of Rome, Rome, Italy; Laboratory of Virology, Department of Public Health and Infectious Diseases, “La Sapienza” University of Rome, Rome, Italy; Department of Pharmacology, IRCCS Policlinico S. Matteo, Pavia, Italy; I.N.M.I. Lazzaro Spallanzani, Rome, Italy; Neonatal Intensive Care Unit, Department of Pediatrics and Child Neuropsychiatry, “Sapienza” University of Rome, V.le Regina Elena 324, 00161 Rome, Italy

**Keywords:** Valganciclovir, Ganciclovir, Cytomegalovirus, Cerebrospinal fluid, Congenital infection

## Abstract

Currently, there is no evidence whether ganciclovir, or its oral prodrug valganciclovir, penetrates into the cerebrospinal fluid of human infants treated for congenital cytomegalovirus infection. Here, we report a case study providing evidence that ganciclovir, administered as valganciclovir, reaches the infant’s cerebrospinal fluid when used at the currently recommended dose for congenital cytomegalovirus infection.

## Background

Ganciclovir (GCV) is the drug of choice for the treatment of congenital cytomegalovirus (CMV) infection. In fact, 6 weeks intravenous GCV therapy, compared with no treatment, has been demonstrated to significantly prevent hearing deterioration [1] and developmental delays [2] in infants with congenital CMV infection involving the central nervous system. Valganciclovir (V-GCV), an oral pro-drug of GCV, nowadays represents a valuable alternative to GCV and, compared to GCV, does not require prolonged hospitalization and catheterization, thus avoiding considerable costs for the Health Care System, and infectious risks for the infants [3]. For both these drugs, cerebrospinal fluid (CSF) penetration has never been demonstrated in congenitally CMV infected infants undergoing antiviral therapy at the currently recommended dosages.

## Case report

A 30-year-old woman had a primary CMV infection in the first trimester of pregnancy (gestation week 9), defined by the presence of immunoglobulin (Ig) M and low avidity IgG. At gestation week 20, CMV DNA was evidenced in the amniotic fluid sample at concentrations of 3.8 × 10^5^ genomic copies (GC)/mL. Intrauterine growth retardation was detected starting gestation week 30. A foetal brain magnetic resonance imaging (MRI), performed two weeks later, was normal. A small for gestational age (<10° centile for gestational age) female infant in good clinical conditions was delivered at 38 weeks gestational age. Congenital CMV infection was confirmed by means of quantitative Polymerase Chain Reaction (qPCR) performed on urine sampled on day of life (dol) 2.

On dol 11, the infant was referred to our centre to undergo a diagnostic workup for congenital CMV infection. Clinical examination, fundus oculi, abdominal and cerebral ultrasound, and auditory brainstem evoked responses were all negative; complete blood count and transaminase levels were in the normal range for the infant’s age. Of note, a brain MRI performed at 1.5 Tesla (Magnetom Avanto, Siemens Medical Solution, Malvern, PA, USA) failed to find any brain lesions. CMV DNA search was performed on urine and blood samples by an automated system for nucleic acid extraction (Nuclisens EasyMag, Biomerieux) and a commercially available TaqMan Real Time Polymerase Chain Reaction kit (ELITech Group S.p.A.) for quantitative CMV DNA amplification. According to our screening procedure, and following informed consent by the parents, a lumbar puncture for cerebrospinal fluid (CSF) CMV DNA search and CSF indices was carried out. CMV DNA was detected in all the samples tested (Table 1).

**Table 1:** **Summary of the laboratory findings obtained on cerebrospinal fluid, blood, and urine samples before (dol 11) and after 25 days of Valganciclovir therapy (dol 45)**

**Findings**	**Dol 11**	**Dol 45**
Cerebrospinal Fluid		
Proteins	67,9 mg/dL	59,0 mg/dL
White Cells	12 cells/μL	9 cells/μL
Red Cells	141 cells/μL	373 cells/μL
CMV-DNA	2.835.371 GC/ml	Negative
GCV	-	0,52 μg/ml
Blood		
CMV-DNA	115.068 GC/ml	821 GC/ml
GCV (trough)	-	0,53 μg/ml
GCV (peak)	-	5,54 μg/ml
Urine		
CMV-DNA	>25.000.000 GC/ml	619.137 GC/ml

See text for the abbreviations.

Since the CMV-DNA search in the CSF was positive, on dol 20 we decided to start antiviral treatment with a commercial oral solution of V-GCV (Valcyte®, Roche S.p.A., Segrate, Italy) at 16 mg/kg/dose twice a day. On dol 21, after the uneventful administration of two doses of V-GCV, the baby was discharged home. The drug dosage was adjusted weekly according to the weight increase. During V-GCV therapy, complete blood count, transaminase, and creatinine levels were monitored three times (on dol 27, 45, and 55 respectively) for possible drug toxicity: the first two blood tests were within the normal range for the patient’s age whereas, on dol 55, a remarkable transaminase increase was detected (Alanine aminotransferase: 328 U/L, Aspartate Aminotransferase: 400 U/L). Due to the onset of liver toxicity presumably related to antiviral therapy, V-GCV was withdrawn on dol 55 (after 35 days of therapy) and never resumed. Fifteen days later, transaminase levels were in the normal range.

CMV DNA qPCR on plasma, urine and CSF samples were again performed on dol 45 (Table 1). At the same time, plasma trough and peak values (one hour before and two hours after V-GCV administration, respectively) and CSF GCV concentration (performed concomitantly with GCV plasma trough sample) were evaluated by means of High-Performance Liquid Chromatography assay with fluorescence detection (Table 1). Ongoing clinical, ophthalmological, audiological and neurological follow-up (by means of the Bayley Scales of Infant Development-III) is normal at twelve months of age.

## Discussion

A randomized, controlled trial, demonstrated that ganciclovir, administered at 6 mg/kg/dose twice a day for 6 weeks intravenously, is able to prevent hearing deterioration [1] and developmental delays [2] in infants with congenital CMV infection involving the central nervous system. V-GCV, an oral pro-drug of ganciclovir that is rapidly converted into ganciclovir after intestinal absorption, reached plasma concentrations comparable to those achieved with administration of intravenous ganciclovir when used at 16 mg/kg/dose twice a day [4]. V-GCV has demonstrated to be well tolerated and is increasingly used because, compared to GCV, it does not require hospitalization and catheterization, thus avoiding considerable costs for the Health Care System and infectious risks for the infants. Moreover, V-GCV seems to be suitable for prolonged home therapy [3].

The evaluation of CSF levels is used to predict brain interstitial fluid concentrations of a drug [5]. Although GCV penetration into the CSF has been demonstrated in some reports dealing with CMV infected adults and children in different clinical situation (an adult suffering renal failure [6], a child with acute lymphoblastic leukemia who exhibited a reduced creatinine clearance [7], and two bone marrow transplanted patients on different ganciclovir regimen therapy [8]), our report provides evidence, for the first time, that GCV, administered as V-GCV, reaches the infant’s CSF when used at the currently recommended dosages for congenital CMV infection [4]. Our GCV plasma trough and peak concentrations (Table 1), detected by means of a validated technique [9], were consistent with those reported in the medical literature [3]; GCV plasma (trough value) and CSF levels (trough value) were overlapping in this case (Table 1), although additional results are needed to establish any significant pharmacokinetic correlation between plasma and CSF GCV concentrations in neonates.

CMV DNA qPCR performed on CSF before and during V-GCV therapy (Table 1) showed a reduced viral load in the CSF, suggestive of effective therapy on brain infection. Although the CSF GCV concentration of 0,52 μg/ml we reported was below the inhibitory concentration (IC) 50 for most CMV clinical isolates [10], it must be taken into account that the CSF sample was drawn concomitantly with the plasma trough sample, thus allowing a GCV CSF peak level over the requested IC 50. Besides, the CSF CMV DNA negativisation cannot be fully explained since the persistence of CMV DNA into the CSF of untreated infants is unknown.

According to published guidelines [11], and in order to prevent possible ongoing brain damage resulting from CMV replication in the infant’s brain, we decided (following the parents’ informed consent) to start V-GCV therapy in this patient given the positive CSF CMV DNA and despite normal CSF indices (Table 1) and neuroimaging. Nonetheless, in our opinion, further studies are necessary to evaluate the need for therapy, if any, in infants with positive CSF CMV DNA and undetectable brain damage.

## Consent

Written informed consent was obtained from the patient’s parents to publish this Case report. A copy of the written consent can be submitted to the Editor-in-Chief of this journal.

## Abbreviations

GCV: Ganciclovir
CMV: Cytomegalovirus
V-GCV: Valganciclovir
CSF: Cerebrospinal fluid
Ig: Immunoglobulin
GC: Genomic copies
MRI: Magnetic Resonance Imaging
qPCR: quantitative Polymerase Chain Reaction
DOL: Day of life
IC: inhibitory concentration

## References

[1] Kimberlin DW, Lin CY, Sánchez PJ, Demmler GJ, Dankner W, Shelton M (2003). Effect of ganciclovir therapy on hearing in symptomatic congenital cytomegalovirus disease involving the central nervous system: a randomized, controlled trial. J Pediatr.

[2] Oliver SE, Cloud GA, Sánchez PJ, Demmler GJ, Dankner W, Shelton M (2009). Neurodevelopmental outcomes following ganciclovir therapy in symptomatic congenital cytomegalovirus infections involving the central nervous system. J Clin Virol.

[3] Stronati M, Lombardi G, Garofoli F, Villani P, Regazzi M (2013). Pharmacokinetics, pharmacodynamics and clinical use of valganciclovir in newborns with symptomatic congenital cytomegalovirus infection. Curr Drug Metab.

[4] Kimberlin DW, Acosta EP, Sánchez PJ, Sood S, Agrawal V, Homans J (2008). Pharmacokinetic and pharmacodynamic assessment of oral valganciclovir in the treatment of symptomatic congenital cytomegalovirus disease. J Infect Dis.

[5] Nau R, Sörgel F, Eiffert H (2010). Penetration of drugs through the blood-cerebrospinal fluid/blood–brain barrier for treatment of central nervous system infections. Clin Microbiol Rev.

[6] Sakamoto H, Hirano M, Nose K, Ueno S, Oki T, Sugimoto K (2013). A case of severe ganciclovir-induced encephalopathy. Case Rep Neurol.

[7] Peyriere H, Jeziorsky E, Jalabert A, Cociglio M, Benketira A, Blayac JP (2006). Neurotoxicity related to valganciclovir in a child with impaired renal function: usefulness of therapeutic drug monitoring. Ann Pharmacother.

[8] Fletcher C, Sawchuk R, Chinnock B, de Miranda P, Balfour HH (1986). Human pharmacokinetics of the antiviral drug DHPG. Clin Pharmacol Ther.

[9] Chu F, Kiang CH, Sung ML, Huang B, Reeve RL, Tarnowski T (1999). A rapid, sensitive HPLC method for the determination of ganciclovir in human plasma and serum. J Pharm Biomed Anal.

[10] McSharry JJ, McDonough A, Olson B, Talarico C, Davis M, Biron KK (2001). Inhibition of ganciclovir-susceptible and -resistant human cytomegalovirus clinical isolates by the benzimidazole L-riboside 1263W94. Clin Diagn Lab Immunol.

[11] Kadambari S, Williams EJ, Luck S, Griffiths PD, Sharland M (2011). Evidence based management guidelines for the detection and treatment of congenital CMV. Early Hum Dev.

